# Acute Facility Management of Blast Injuries In Low- and Middle-Income Countries: A Systematic Review and Meta-Analysis

**DOI:** 10.1017/S1049023X25101222

**Published:** 2025-06

**Authors:** Charlotte M. Roy, Stephanie C. Garbern, Pryanka Relan, Corey B. Bills, Megan L. Schultz, Alex H. Wang, Hayley E. Severson, Braden J. Hexom, Sean M. Kivlehan

**Affiliations:** 1. Department of Emergency Medicine, Keck School of Medicine of University of Southern California, Los Angeles, California USA; 2. Department of Emergency Medicine, Warren Alpert Medical School of Brown University, Providence, Rhode Island USA; 3. WHO Health Emergencies Program, World Health Organization, Geneva, Switzerland; 4. Department of Emergency Medicine, University of Colorado School of Medicine, Aurora, Colorado USA; 5. Department of Pediatrics, Medical College of Wisconsin, Milwaukee, Wisconsin USA; 6. Department of Emergency Medicine, St. Charles Health System, Bend, Oregon USA; 7. Medical College of Wisconsin Libraries, Milwaukee, Wisconsin USA; 8. Department of Emergency Medicine, Rush University Medical Center, Chicago, Illinois USA; 9. Department of Emergency Medicine, Brigham and Women’s Hospital, Boston, Massachusetts USA; 10. Harvard Humanitarian Initiative, Cambridge, Massachusetts USA

**Keywords:** blast injuries, developing countries, disaster medicine, emergency medicine, international health

## Abstract

**Introduction:**

Blast injuries can occur by a multitude of mechanisms, including improvised explosive devices (IEDs), military munitions, and accidental detonation of chemical or petroleum stores. These injuries disproportionately affect people in low- and middle-income countries (LMICs), where there are often fewer resources to manage complex injuries and mass-casualty events.

**Study Objective:**

The aim of this systematic review is to describe the literature on the acute facility-based management of blast injuries in LMICs to aid hospitals and organizations preparing to respond to conflict- and non-conflict-related blast events.

**Methods:**

A search of Ovid MEDLINE, Scopus, Global Index Medicus, Web of Science, CINAHL, and Cochrane databases was used to identify relevant citations from January 1998 through July 2024. This systematic review was conducted in adherence with PRISMA guidelines. Data were extracted and analyzed descriptively. A meta-analysis calculated the pooled proportions of mortality, hospital admission, intensive care unit (ICU) admission, intubation and mechanical ventilation, and emergency surgery.

**Results:**

Reviewers screened 3,731 titles and abstracts and 173 full texts. Seventy-five articles from 22 countries were included for analysis. Only 14.7% of included articles came from low-income countries (LICs). Sixty percent of studies were conducted in tertiary care hospitals. The mean proportion of patients who were admitted was 52.1% (95% CI, 0.376 to 0.664). Among all in-patients, 20.0% (95% CI, 0.124 to 0.288) were admitted to an ICU. Overall, 38.0% (95% CI, 0.256 to 0.513) of in-patients underwent emergency surgery and 13.8% (95% CI, 0.023 to 0.315) were intubated. Pooled in-patient mortality was 9.5% (95% CI, 0.046 to 0.156) and total hospital mortality (including emergency department [ED] mortality) was 7.4% (95% CI, 0.034 to 0.124). There were no significant differences in mortality when stratified by country income level or hospital setting.

**Conclusion:**

Findings from this systematic review can be used to guide preparedness and resource allocation for acute care facilities. Pooled proportions for mortality and other outcomes described in the meta-analysis offer a metric by which future researchers can assess the impact of blast events. Under-representation of LICs and non-tertiary care medical facilities and significant heterogeneity in data reporting among published studies limited the analysis.

## Introduction

Blast injuries are unpredictable and catastrophic events resulting in serious injury or death. Explosive weapons cause tens of thousands of annual injuries and deaths in these contexts.^1^ As the number of people living in conflict zones rises, blast events related to warfare or terrorism impact a growing population.^2^ Blast injuries also often occur outside of the context of conflict. Non-conflict-related explosions, including the 2020 Beirut port explosion and the 2021 Freetown fuel tanker explosion, account for some of the largest mass-casualty events in the past ten years.^3,4^

The term “blast injury” refers to any traumatic injury resulting from an explosion, itself defined as the rapid expansion of gas. This can occur intentionally or unintentionally and by a multitude of mechanisms, including military devices (such as munitions and mines), improvised explosive devices (IEDs), and accidental detonation of chemical or petroleum stores. Explosions cause harm via several mechanisms, including penetrating trauma, blunt trauma, and burns. The exact nature of the injuries caused by a blast event depends on numerous factors, including the mechanism of the explosion, the surrounding environment, the position of the explosive, and whether the event occurred in an open or enclosed space.^5^

Injury due to a blast has traditionally been divided into four categories: primary, secondary, tertiary, and quaternary.^6^ Primary blast injury results from the blast wave itself, with injuries most often involving the lung, tympanic membrane, brain, and hollow viscus organs. Secondary blast injury refers to trauma from fragments propelled by the blast wave, such as shrapnel. Tertiary blast effects describe injuries due to displacement of the human body or of large objects into the body. Quaternary effects include burns, inhalation injury, and chemical exposure resulting from a blast.

Blast injuries disproportionately affect people in low- and middle-income countries (LMICs), where poverty acts as both a driver and consequence of conflict.^2^ Morbidity and mortality from blast events may be higher in these settings due to fewer resources to manage complex injuries and mass-casualty incidents.^7^ Low-income countries (LICs) bear a proportionally greater burden of blast-related trauma. For example, 23.3% of all explosive terrorist attacks from 1998 through 2020 occurred in LICs, even though these countries represented only six percent to nine percent of the world’s population during the same time period.^8,9^ Furthermore, landmine injuries almost exclusively affect people living in LMICs.^10^ Lower income countries are also more likely to have insufficient safety regulations resulting in non-conflict-related explosive events, such as chemical or petroleum explosions.^11^

The study of blast injuries presents a conundrum: although blast injuries occur disproportionately in LMICs, disparities in the availability of research funding and training pose limitations to the ability of researchers in these settings to conduct and publish studies. In addition, explosions are low frequency, high consequence events that are inherently unexpected for most victims, making it difficult for researchers to prospectively plan experimental or quasi-experimental studies. To the authors’ knowledge, this is the first systematic review of blast injuries specifically focused on LMICs.

The goal of this systematic review is to provide an overview of the literature on the acute facility management of blast injuries in LMICs. A deeper understanding of this subject will assist hospitals and local and international organizations preparing to respond to both conflict- and non-conflict-related blast events, including mass-casualty events. This systematic review also aims to highlight disparities in the available evidence and areas needing further study.

## Methods

This systematic review was conducted in collaboration with the Global Emergency Medicine Literature Review (GEMLR) group. The authors adhered to the Preferred Reporting Items for Systematic Reviews and Meta Analyses (PRISMA) guidelines (Appendix S1; available online only).^12^ The review protocol was registered on PROSPERO on November 6, 2023 (CRD42023474931). The published study protocol is available in Appendix S2 (available online only). All data were previously published and de-identified, therefore this study was exempt from Institutional Review Board review.

A systematic search of the literature was developed and conducted by a medical librarian (HS) with input from the research team (Appendix S3; available online only). The search strategy was initially created for Ovid MEDLINE (US National Library of Medicine, National Institutes of Health; Bethesda, Maryland USA) using a combination of Medical Subject Heading (MeSH) terms, keywords, and phrases related to facility-based management of blast injuries. Search terms for LMICs were based on Cochrane LMIC filters and current World Bank (Washington, DC USA) classification.^13^ The strategy was translated using advanced search techniques relevant to each additional database: Scopus (Elsevier; Amsterdam, Netherlands); Web of Science Core Collection (Clarivate Analytics; London, United Kingdom); CINAHL (EBSCO Information Services; Ipswich, Massachusetts USA); Cochrane Central Register of Controlled Trials (*Wiley; Hoboken, New Jersey USA)*; Cochrane Database of Systematic Reviews (*Wiley; Hoboken, New Jersey USA)*; and Global Index Medicus (World Health Organization [WHO]; Geneva, Switzerland). The search was completed on November 20, 2023 and included citations published from January 1, 1998 through November 20, 2023. The search was re-run on July 12, 2024 and included additional citations published from January 1, 1998 through July 12, 2024. Articles published in English, French, or Spanish were included. Gray literature and conference proceedings were excluded.

Abstracts were uploaded in Covidence (Veritas Health Innovation; Melbourne, Australia) and duplicates were removed. Each abstract was screened by two independent reviewers (CR, MS, CB, AW) with discrepancies resolved by a third reviewer. All articles related to acute facility management of blast injuries, defined as emergency department (ED) and acute and early in-patient management, were included. Excluded were studies: conducted in a high-income country (HIC); related to nuclear explosions, cell phone explosions, or fireworks; exclusively of combatants; pertaining solely to ocular or tympanic injuries or mental health effects; focused on non-acute interventions or prehospital care. Articles on multiple traumatic mechanisms that did not disaggregate data specific to blast injuries were also excluded. Disaggregation of data by blast injury classification (eg, primary versus secondary) was not required for inclusion. For all abstracts screened in, two independent reviewers read the full-text article to assess for inclusion with discrepancies resolved by a third reviewer. CR, MS, CB, and AW conducted a risk of bias assessment of all included studies using the Newcastle-Ottawa Scale for cross-sectional studies.

Data pertaining to study setting, study design, blast mechanism, population, injury epidemiology, acute interventions, and mass-casualty preparedness were extracted and analyzed descriptively by CR using Microsoft Excel (Microsoft Corp.; Redmond, Washington USA). The data extraction template is shown in Supplementary Table S1 (available online only). Regarding blast mechanism, in many cases, it is impossible to determine if an injury occurred due to an IED or military munitions, as both can impact civilians living in conflict zones. To avoid making assumptions about the origin of the explosive device, IEDs and military munitions were combined in a single category of blast mechanism during data extraction. Injuries were grouped by anatomic region with the exception of burns and inhalation burns, categorized separately. Acute interventions included medications, surgery, respiratory support, and burn care. Studies were classified geographically by WHO region.^14^ Country income level was assigned according to World Bank classification.^15^

A meta-analysis of patient outcomes and resource utilization was performed for a subset of articles using StataNow/SE 18.5 (StataCorp; College Station, Texas USA). Case reports, studies focused on a specific injury type, studies without mortality data, and studies with mortality data not disaggregated by traumatic mechanism were excluded from meta-analysis. Studies that included duplicate data already presented by another article were also excluded. When studies contained overlapping data sets, the study with (1) more rigorous methodology, (2) a larger total number of participants, or (3) more complete mortality-related data was included (in order of priority).

Pooled (mean) outcomes and 95% confidence intervals (CI) were calculated using a random-effects Bayesian model. Effect size was calculated using Freeman-Tukey transformation. Leave-one-out analysis and cumulative analysis were performed to evaluate outliers and possible small-study effects. Outcomes included total hospital mortality (among all patients presenting to the hospital, including those who died in the ED) and in-patient mortality (among only admitted patients). Patients who were deceased on arrival to the ED were excluded from both calculations. Meta-analysis was also performed for proportions of hospital admission, intensive care unit (ICU) admission, intubation and mechanical ventilation, and emergency surgery.

Secondary outcomes included stratifying the findings to explore potential influences of study country income level, mechanism of blast, and hospital setting on the outcome of mortality. Analysis was performed using a random-effects model due to known heterogeneity across studies. Secondary outcomes are presented as unadjusted 95% confidence intervals and should be used for hypothesis generation only.

## Results

**Figure 1.**
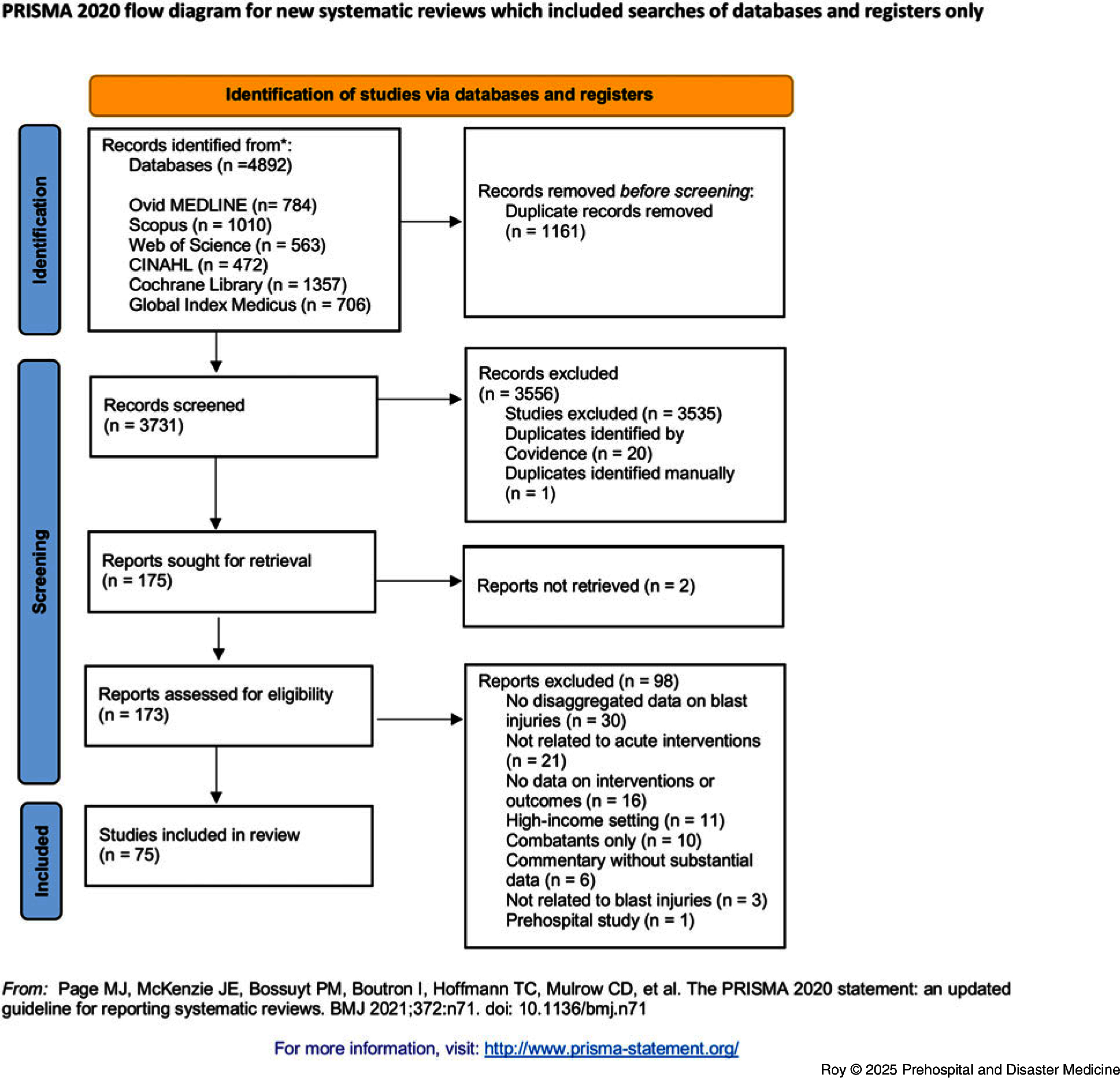
PRISMA Flow Chart. Abbreviation: PRISMA, Preferred Reporting Items for Systematic Reviews and Meta Analyses.

The search retrieved 4,892 articles, of which 1,161 were duplicates (Figure 1). Reviewers screened 3,731 titles and abstracts and 173 full texts. Seventy-five articles were included for analysis (Table 1 and Table 2). A complete list of references for all included studies is available in Appendix S4 (available online only).

**Table 1.** Characteristics of Included Studies

Study characteristic N (%)	
**Blast mechanism N (%)**	
IED/Munitions	46 (61.3)
Chemical	13 (17.3)
Industrial	4 (5.3)
Mines	3 (4.0)
Petroleum	3 (4.0)
Other	3 (4.0)
Multiple	2 (2.7)
Unspecified	1 (1.3)
**Hospital Setting N (%)**	
Tertiary Care	45 (60.0)
Combat/Military	14 (18.7)
Multiple	6 (8.0)
Community Hospital	5 (6.7)
Field Hospital	2 (2.7)
Other	3 (4.0)
**Study design N (%)**	
Cross-Sectional	58 (77.3)
Case Series	6 (8.0)
Case Report	5 (6.7)
Cohort	3 (4.0)
Non-Randomized Experimental	1 (1.3)
Other	2 (2.7)
**Country N (%)**	
Pakistan	11 (14.7)
China	9 (12.0)
Iraq	8 (10.7)
Turkey	8 (10.7)
Lebanon	7 (9.3)
Afghanistan	6 (8.0)
Nigeria	4 (5.3)
India	3 (4.0)
Others	20 (26.7)
**WHO Region**	
EMR	38 (50.7)
EUR	12 (16.0)
WPR	11 (14.7)
SEAR	6 (8.0)
AFR	5 (6.7)
AMR	3 (4.0)
**World Bank Income Level**	
Upper Middle-Income	33 (44.0)
Lower Middle-Income	31 (41.3)
Low-Income	11 (14.7)

Abbreviations: IED, improvised explosive device; WHO, World Health Organization.

**Table 2.** List of Included Studies

Study ID	Country	Design	Blast Mechanism	Year(s) Data Collected	N	Included in Mortality Meta-Analysis
Al-Hajj 2023a	Lebanon	Cross-Sectional	Chemical	2020	3,237	Y
Al-Hajj 2023b	Lebanon	Cross-Sectional	Chemical	2020	791	N^a^
Amole 2021	Nigeria	Cross-Sectional	IED/Munitions	2014	50	Y
Arafat 2017	Syria	Cross-Sectional	IED/Munitions	2012-2013	183	N^b^
Arslan 2022	Somalia	Cross-Sectional	IED/Munitions	2014-2021	1,073	Y
Atici 2020	Turkey	Case Report	IED/Munitions	NA	1	N^c^
Atreya 2016	Nepal	Case Report	Other	NA	1	N^c^
Bendinelli 2009	Cambodia	Cross-Sectional	Mines	2003-2006	356	Y
Benzar 2024	Ukraine	Case Series	IED/Munitions	2022	8	Y
Biancolini 1999	Argentina	Cross-Sectional	IED/Munitions	1994	84	Y
Bilukha 2007	Russia	Cross-Sectional	Mines	1994-2005	3,021	N^d^
Brisson 2011	Afghanistan	Case Report	Industrial	NA	1	N^c^
Cardi 2019	Afghanistan	Cross-Sectional	IED/Munitions; Mines	2006-2016	267	N^b^
Celik 2018	Turkey	Cross-Sectional	IED/Munitions	2015-2016	51	Y
Chai 2007	China	Case Series	Other	2005	5	Y
Demirel 2021	Somalia	Cross-Sectional	IED/Munitions	2017	252	Y
Deshpande 2007	India	Other: Correspondence	IED/Munitions	2006	76	Y
Diallo 2020	Burkina Faso	Cross-Sectional	Industrial	2016-2018	30	N^b^
Dominguez 2011	Iraq	Case Series	IED/Munitions	2007	11	N^b^
Dong 2021	China	Other: Narrative	Chemical	2015	322	N^d^
ElZahran 2022	Lebanon	Cross-Sectional	Chemical	2020	349	N^a^
Fadeyibi 2009	Nigeria	Cross-Sectional	Petroleum	2006	90	Y
Fadeyibi 2011	Nigeria	Cohort Study	Petroleum	1999-2007	48	N^a^
Gebran 2022	Lebanon	Cross-Sectional	Chemical	2020	1,818	N^a^
Guo 2015	China	Cross-Sectional	Chemical	2015	233	Y
Hallal 2021	Lebanon	Cross-Sectional	Chemical	2020	378	N^a^
Harrison 2009	Iraq	Cross-Sectional	IED/Munitions	2006	167	N^d^
Hoz 2021	Iraq	Cross-Sectional	IED/Munitions	2009	56	N^b^
Inwald 2014	Afghanistan	Cross-Sectional	IED/Munitions	2011-2012	52	N^b^
Jimenez 2019	Colombia	Cross-Sectional	IED/Munitions	2003	63	Y
Karaca 2024	Syria	Cross-Sectional	IED/Munitions	2021	245	Y
Khan 2012	Pakistan	Cross-Sectional	IED/Munitions	2009-2010	154	N^b^
Kumar 2010	India	Case Series	Industrial	2003	6	Y
Li 2015	China	Cross-Sectional	Chemical	2015	298	Y
Malik 2006	Pakistan	Cross-Sectional	IED/Munitions	2004	88	N^d^
Mansour 2021	Lebanon	Cross-Sectional	Chemical	2020	8,606	N^a^
Martinovic 2008	Bosnia and Herzegovina	Cross-Sectional	IED/Munitions	1993-1994	1,022	Y
McGuigan 2007	Iraq	Cross-Sectional	IED/Munitions	2004	22	Y
Muhammad-Umair 2013	Pakistan	Cross-Sectional	IED/Munitions	Unspecified	16	N^b^
Nerlander 2021	Iraq	Cross-Sectional	IED/Munitions	2017	1,595	Y
Ojo 2016	Nigeria	Cross-Sectional	IED/Munitions	2010-2012	12	N^b^
Ozluer 2021	Turkey	Cross-Sectional	IED/Munitions	2016-2017	100	Y
Pan 2023	China	Case Report	IED/Munitions	NA	1	N^c^
Parker 2005	Iraq	Cross-Sectional	IED/Munitions	2003	31	N^d^
Parr 2003	Cambodia	Cross-Sectional	Mines	2001	14	N^b^
Pasha 2015	Pakistan	Cross-Sectional	IED/Munitions	1990-2012	22	N^b^
Paydar 2012	Iran	Cross-Sectional	IED/Munitions	2008	202	Y
Raeisi 2019	Iran	Cross-Sectional	Industrial	2017	55	N^d^
Ratnayake 2022	Sri Lanka	Cross-Sectional	IED/Munitions	2019	263	N^d^
Rodoplu 2004	Turkey	Cross-Sectional	IED/Munitions	2003	253	N^d^
Rodoplu 2005a	Turkey	Cross-Sectional	IED/Munitions	2003	76	Y
Rodoplu 2005b	Turkey	Cross-Sectional	IED/Munitions	2003	200	Y
Salinas 2010	Iraq	Cross-Sectional	IED/Munitions	2008	18	N^b^
Sanjuan 2016	Colombia	Non-Randomized Experimental Study	Unspecified	2012-2016	35	Y
Schauer 2018	Iraq, Afghanistan	Cross-Sectional	IED/Munitions	2007-2016	1,462	N^d^
Shah 2015	Pakistan	Cross-Sectional	IED/Munitions	2013	66	Y
Singh 2012	India	Case Series	IED/Munitions	Unspecified	2	N^b^
Sockeel 2008	Afghanistan	Cross-Sectional	IED/Munitions	2007	14	Y
Spagnolello 2022	Afghanistan	Cross-Sectional	IED/Munitions	2021	84	Y
Suljevic 2002	Bosnia and Herzegovina	Cross-Sectional	IED/Munitions	1995	94	Y
Sultan 2018	Pakistan	Cross-Sectional	IED/Munitions	2016	75	Y
Tunthanathip 2018	Thailand	Cohort Study	IED/Munitions	2009-2017	70	N^b^
Umer 2009	Pakistan	Cross-Sectional	IED/Munitions	2002, 2004	32	Y
Uygur 2008	Turkey	Cross-Sectional	Other	2006	7	Y
Vassallo 2005	Kosovo	Cross-Sectional	IED/Munitions	2001	21	N^d^
Yammine 2023	Lebanon	Cross-Sectional	Chemical	2020	159	N^a^
Yasin 2012	Pakistan	Cross-Sectional	IED/Munitions	2007-2010	1,296	Y
Yu 2016	China	Cross-Sectional	Chemical	2015	75	Y
Zafar 2005	Pakistan	Case Series	IED/Munitions	2002	23	Y
Zafar 2011	Pakistan	Cross-Sectional	IED/Munitions	2009, 2010	124	Y
ZafarIqbal 2007	Pakistan	Cross-Sectional	IED/Munitions	2002-2003	48	Y
Zengin 2015	Turkey	Cross-Sectional	Petroleum	2014	69	Y
Zhang 2015	China	Case Report	Chemical	2015	1	N^c^
Zhang 2018	China	Cross-Sectional	Chemical	2015	970	Y
Zheng 2020	China	Cohort Study	Multiple	1997-2017	28	N^b^

Abbreviation: IED, improvised explosive device.

Reason for exclusion from mortality meta-analysis:

a Data duplicated in another study.

b Specific injury type.

c Case report.

d No mortality data or mortality data not disaggregated.

### Study Setting

**Figure 2.**
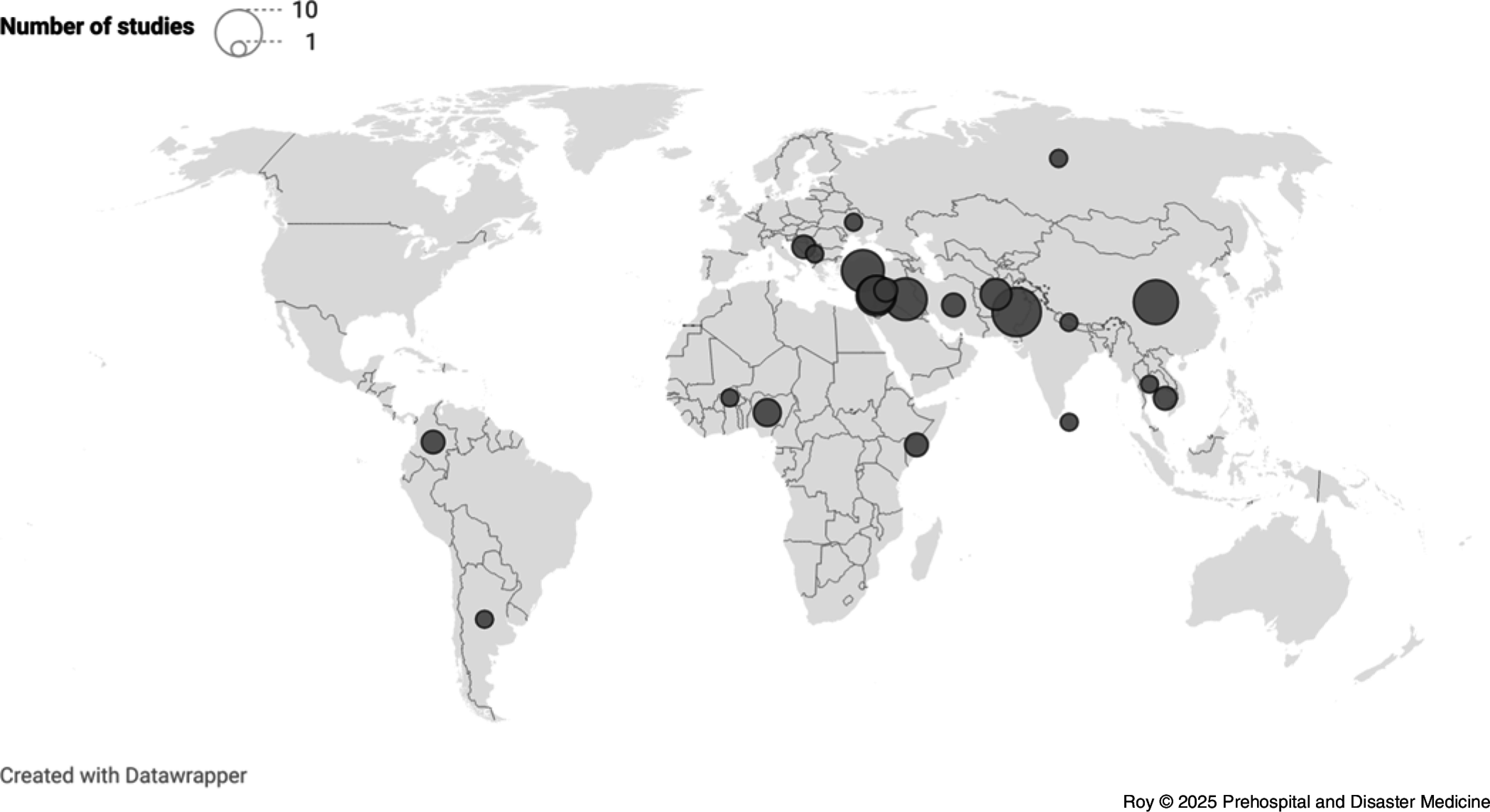
Geographic Distribution of Studies.

A total of 75 articles from 22 countries were included in the final analysis (Figure 2). Roughly one-half (50.7%) of included studies were conducted in the Eastern Mediterranean Region. Other regions represented were the European Region (16.0%), Western Pacific Region (14.7%), South-East Asian Region (8.0%), African Region (6.7%), and Region of the Americas (4.0%). Using World Bank income level classification, most studies (85.3%) were from middle-income countries (44.0% upper-middle, 41.3% lower-middle). Only 14.7% of included articles came from LICs. The majority (60.0%) of studies were conducted in tertiary care hospitals. Less common were studies from combat or military hospitals (18.7%), community hospitals (6.7%), or field hospitals (2.7%).

### Study Design

Most articles (81.3%) were cross-sectional or cohort studies. Case series (8.0%) and case reports (6.7%) were less common. Only one study (1.3%) used a non-randomized experimental study design, and there were no randomized controlled trials.

### Blast Mechanism

The mechanism of the blast event was most often an IED or military munitions (61.3%). Other blast mechanisms studied were chemical (17.3%), industrial (5.3%), mines (4.0%), and petroleum (4.0%). A minority (22.7%) of articles included multiple kinds of traumatic mechanisms, such as trauma due to gunshot wounds or road traffic accidents that occurred either in the same or unrelated incident as the blast event.

### Population

The number of patients ranged from one to 8,606, with a mean of 415 and a median of 75. Exclusively civilian populations were the focus of nearly three-quarters of articles (72.0%). Patients were a mix of civilians and combatants in 13.3%. The presence of combatants was unspecified in 14.7% of articles. The included age range was 26.7% exclusively adult, 8.0% exclusively pediatric (<18 years old), 45.3% both adult and pediatric, and 20.0% unspecified.

### Injury Epidemiology

Among all articles, 87.7% described victims with orthopedic injuries. The next most commonly mentioned injury types were gastrointestinal (64.9%), thoracic (63.2%), and neurologic (61.4%). Also discussed were head and neck (56.1%), burn (42.1%), ocular (29.8%), inhalation burn (15.8%), and genitourinary (12.3%) injuries. Approximately one-quarter (28.0%) of articles focused exclusively on injuries to a specific anatomic region, most often brain injuries (5.33%), extremity injuries (5.33%), or burns (5.33%).

### Acute Interventions

Triage was mentioned in 42.7% of all articles, with six articles (8.0%) identifying the specific triage tool used. Triage tools included Simple Triage and Rapid Treatment (START; 66.7%) and triage sieve (33.3%). The administration of intravenous (IV) fluids, IV antibiotics, and blood transfusions was referenced in 24.0%, 25.3%, and 28.0% of studies, respectively.

In terms of respiratory support, 29.3% of articles described the use of intubation and mechanical ventilation. Only one article (1.3%) discussed using non-invasive ventilation techniques. Few articles (6.7%) referenced the use of simple oxygen therapy, such as nasal cannula or face mask. Other respiratory interventions described included tracheostomy (1.3%) and bronchoscopy (1.3%). The majority (62.7%) of articles noted the availability of an ICU. A few articles (2.7%) stated that ICU care was not available, while others (34.7%) did not specify.

Emergency surgical interventions (defined as surgeries occurring within 24 hours of patient arrival) were common. The most frequently mentioned type of surgery was orthopedic limb surgery, such as fracture repair or amputation, described in 46.7% of included articles. Approximately one-quarter (24.0%) of articles mentioned the use of chest tube thoracostomy. Other categories of surgical intervention included abdominal surgery (40.0%), neurosurgery (32.0%), head and neck surgery (18.7%), and cardiothoracic surgery (12.0%).

A number of articles described the availability of specialty care for burn patients, with 13.3% noting consultation of a burn specialist as part of patients’ treatment. A small number (6.7%) of articles mentioned the use of escharotomy in burn patients.

### Mass-Casualty Preparedness

Approximately one-half (49.3%) of included articles presented analysis of a single mass-casualty event. Among the 37 articles, 67.6% specified the use of a triage system, and 27.0% described the availability of surge staffing for mass-casualty incidents. One study (2.7%) noted the lack of surge staffing, while the remainder (70.3%) did not specify if surge staffing was available. Most (86.5%) articles pertaining to a mass-casualty event identified the availability of ICU-level care, while 13.5% did not specify.

### Meta-Analysis

A meta-analysis was performed on a subset of 43 articles (Table 3). Due to variability in data reporting across studies, each pooled figure was calculated from a different but overlapping subset of studies. Studies excluded from mortality analysis and the reasons for exclusion are indicated in Table 2.

**Table 3.** Meta-Analysis of Resource Utilization and Mortality

Variable	Mean Proportion (0-1)	95% Confidence Interval	Range	N of Studies Reporting Outcome
In-Patient mortality	0.095	(0.046 to 0.156)	0.0-74.4	29
Total Hospital Mortality	0.074	(0.034 to 0.124)	0.0-49.3	26
Hospital Admission	0.521	(0.376 to 0.664)	13.4-100.0	25
ICU Admission ^a^	0.200	(0.124 to 0.288)	4.5-70.1	14
Emergency Surgical Intervention ^a^	0.380	(0.256 to 0.513)	12.5-78.6	11
Intubation and Mechanical Ventilation ^a^	0.138	(0.023 to 0.315)	4.0-85.7	8

a Calculated as a proportion of admitted patients.

The overall proportion of in-patient mortality (in-patient deaths/total number of patients admitted) was 9.5% (95% CI, 4.6% to 15.6%; Figure 3a). In-patient mortality differed significantly by blast mechanism, with blasts secondary to petroleum (61.6%; 95% CI, 36.4% to 84.0%) having higher mortality compared to other blast mechanisms. There was no significant difference in the proportion of in-patient mortality based on the hospital setting or study country income level. A leave-one-out analysis indicated that the omission of Fadeyibi 2009 would result in a slightly lower overall proportion of in-patient mortality when compared with other studies.

**Figure 3a.**
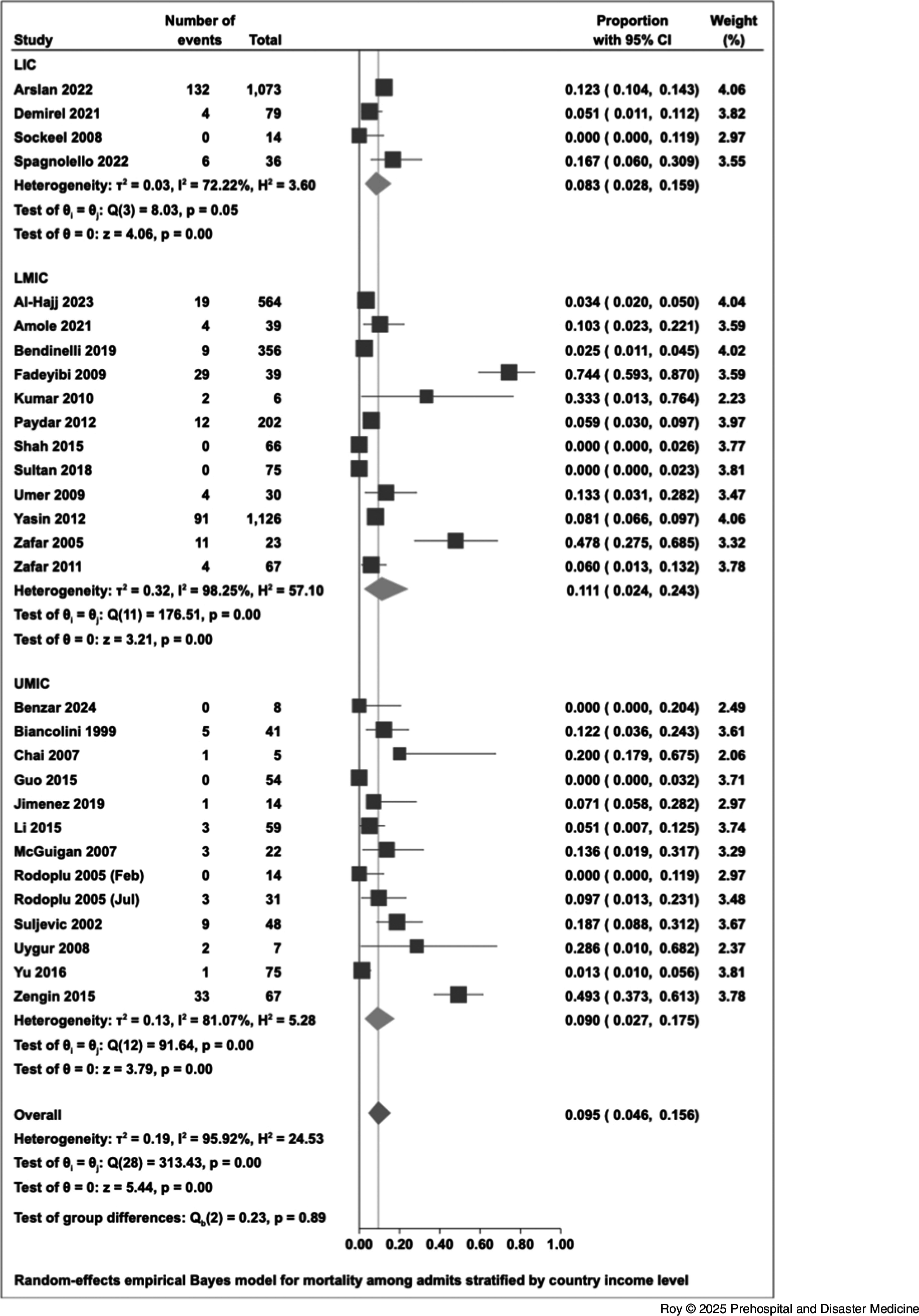
Forest Plot for In-Patient Mortality Stratified by Country Income Level.

The overall proportion of total hospital mortality (ED and in-patient deaths/total number of hospital visits) was 7.4% (95% CI, 3.4% to 12.4%; Figure 3b). Total hospital mortality differed significantly by blast mechanism and by hospital setting. Studies of blasts due to petroleum had significantly higher total hospital mortality (40.4%; 95% CI, 24.4% to 57.4%). Total hospital mortality was significantly lower for studies classified as having multiple hospital settings, of which there was one article (Al Hajj 2023a: 0.6%; 95% CI, 0.3% to 0.9%). Total hospital mortality did not differ significantly by study country income level. Leave-one-out analysis indicated that the omission of Fadeyibi 2009, Zafar 2005, or Zengin 2015 would decrease the overall proportion of total hospital mortality by one percent.

**Figure 3b.**
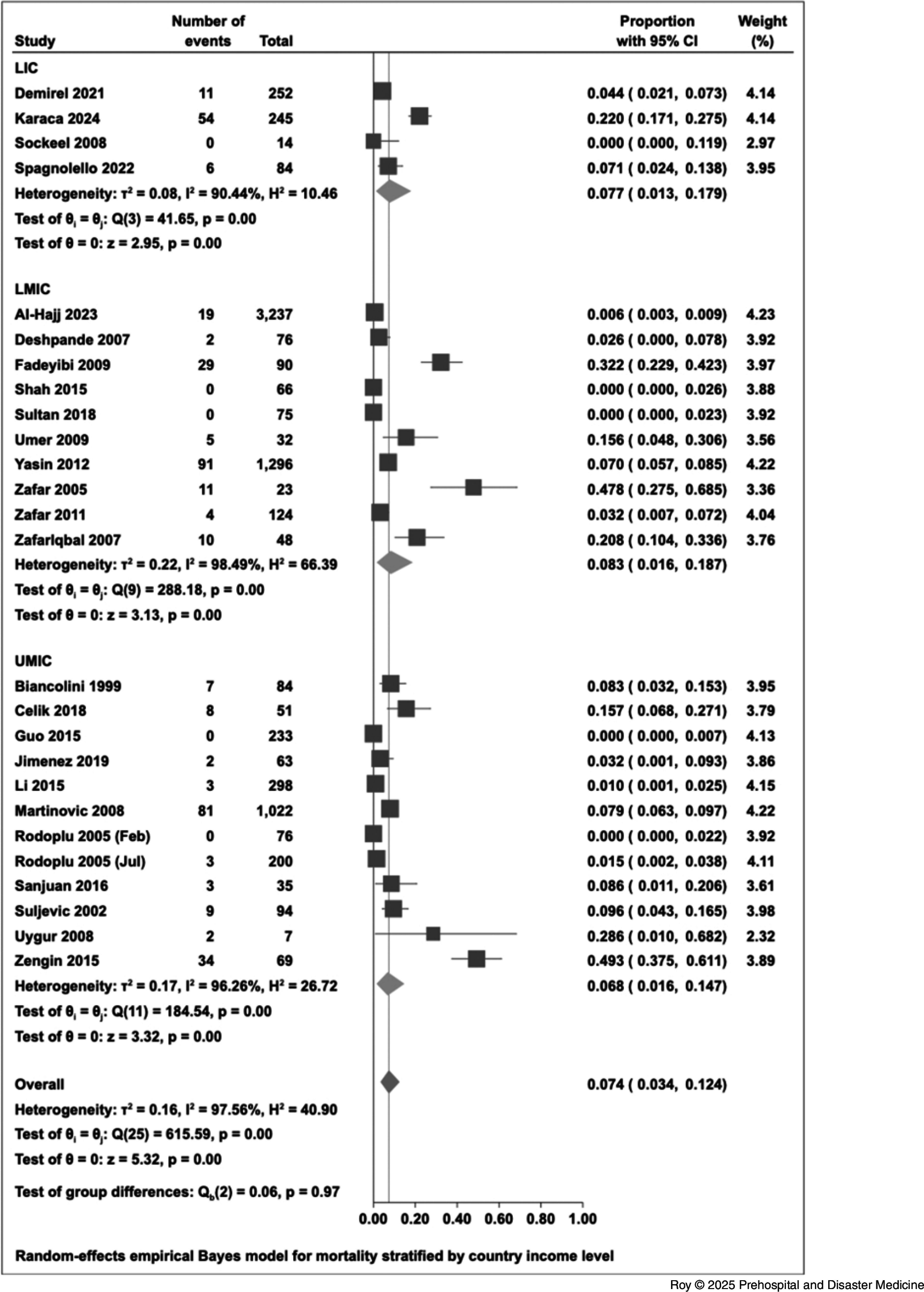
Forest Plot for Total Hospital Mortality Stratified by Country Income Level.

For both in-patient mortality and total hospital mortality, cumulative analysis showed that studies with a smaller number of in-patient admissions had higher overall mortality compared with studies with a larger number of admissions. This is suggestive of small-study effects. Heterogeneity among studies was considerable.

**Figure 4.**
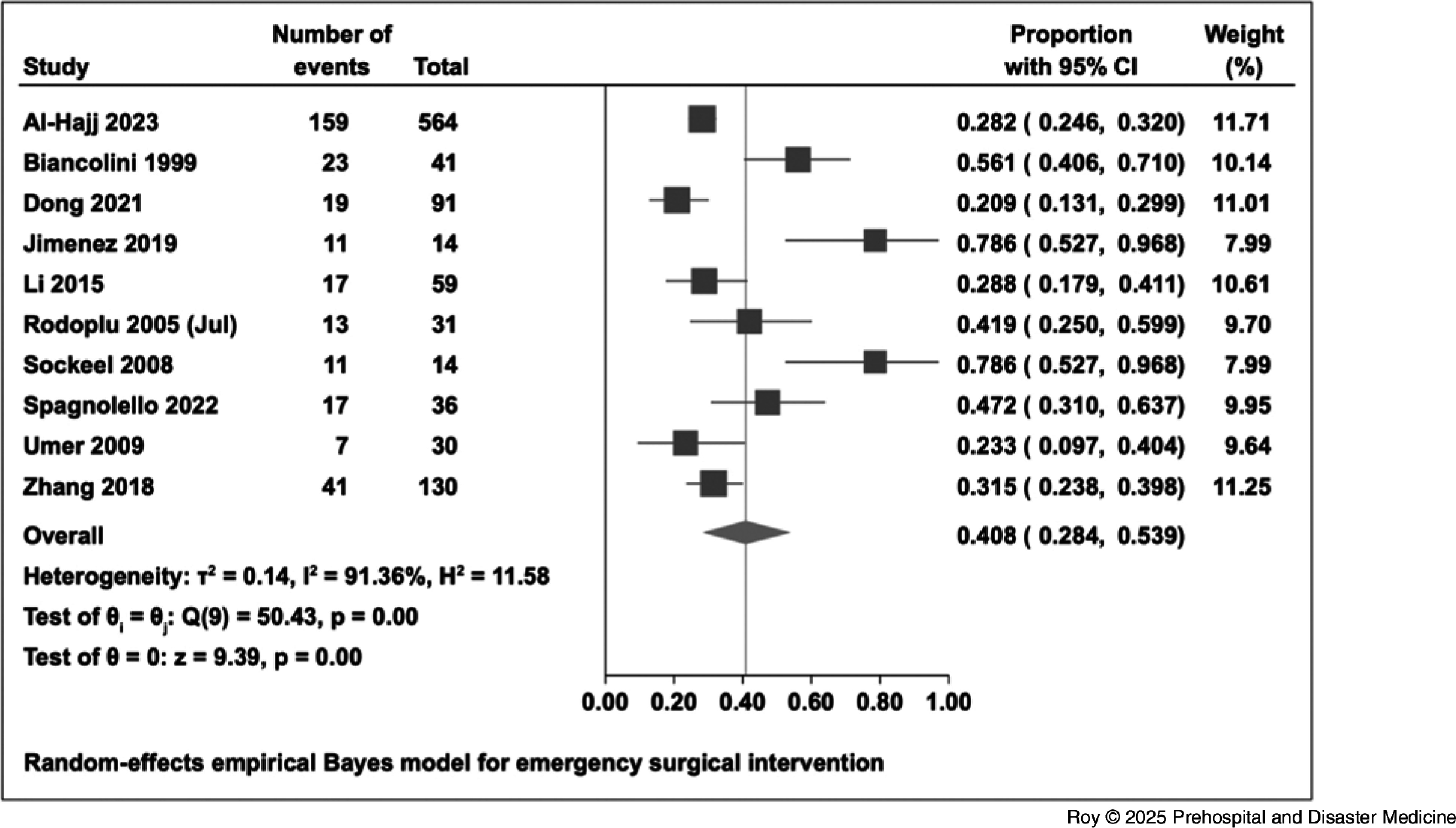
Forest Plot for Emergency Surgical Intervention.

**Figure 5.**
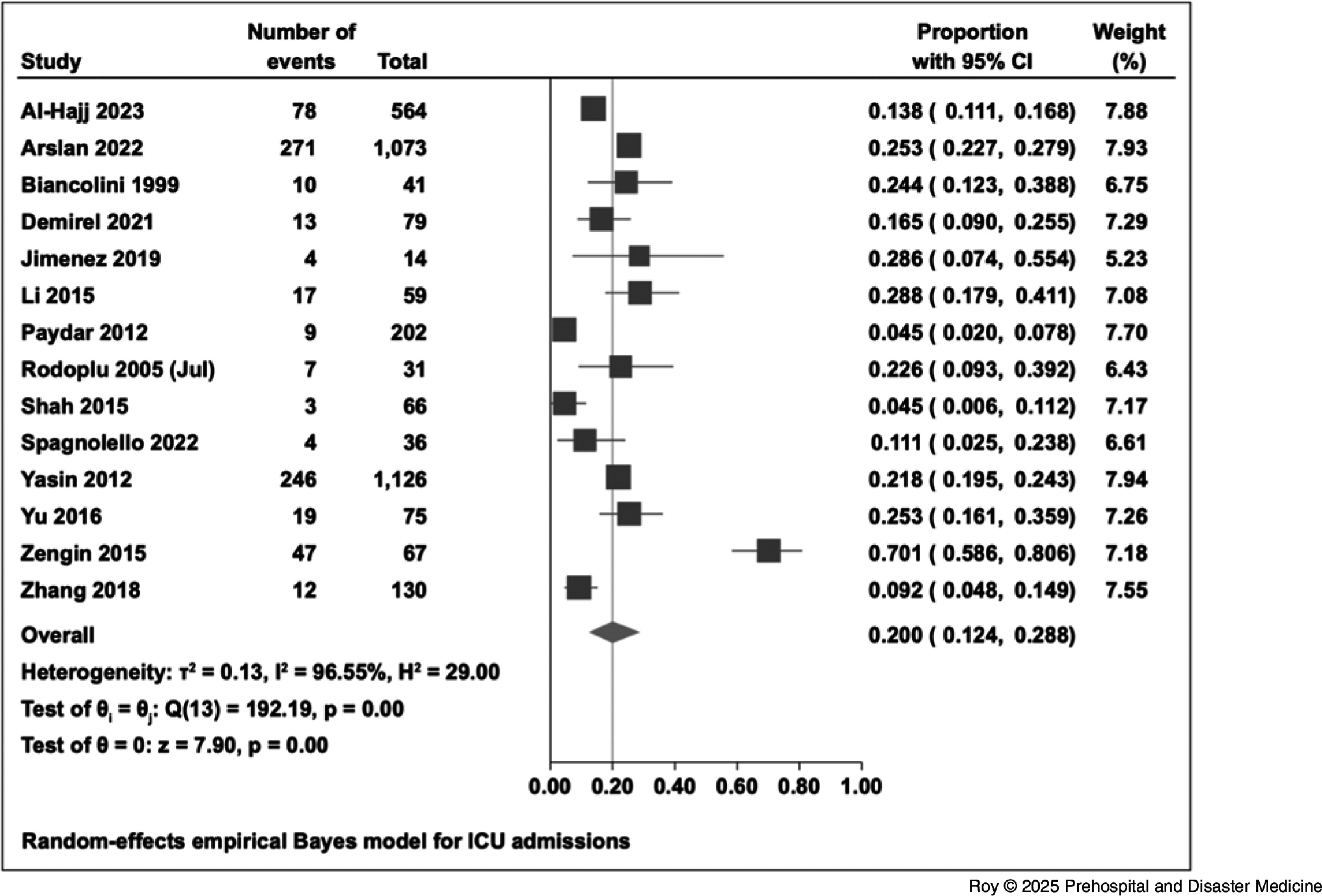
Forest Plot for ICU Admissions. Abbreviation: ICU, intensive care unit.

**Figure 6.**
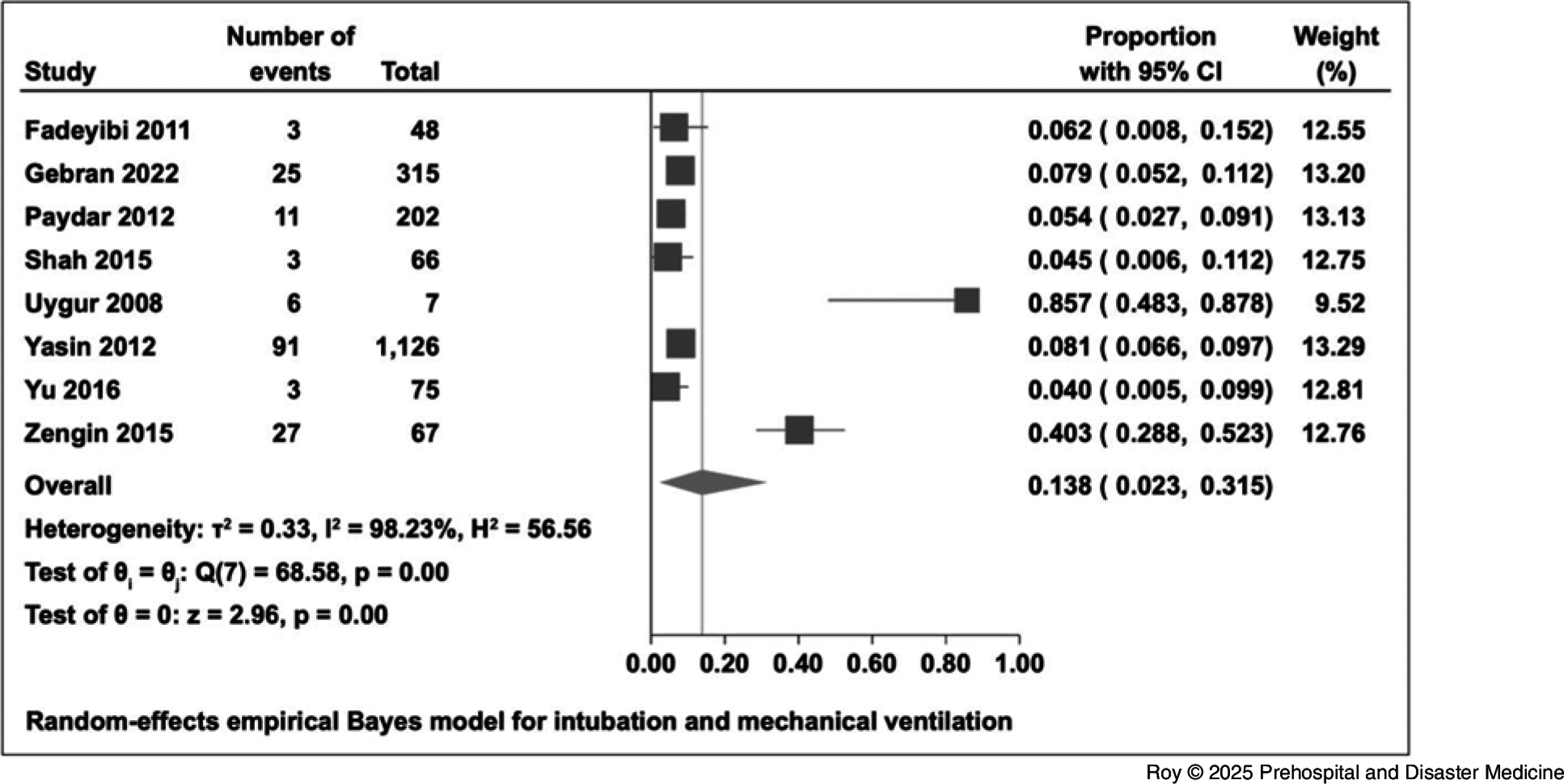
Forest Plot for Intubation and Mechanical Ventilation.

The overall proportion of hospital admissions was 52.1% (95% CI, 37.6% to 66.4%). The proportion of emergency surgical intervention among in-patients was 38.0% (95% CI, 25.6% to 51.3%; Figure 4). The proportion of in-patients who were admitted to an ICU was 20.0% (95% CI, 12.4% to 28.8%; Figure 5). For intubation and mechanical ventilation, the overall proportion was 13.8% (95% CI, 2.3% to 31.5%; Figure 6).

### Risk Of Bias

Based on the Newcastle-Ottawa scale for cross-sectional studies, 59 studies were determined to be at moderate risk of bias. Thirteen studies were high risk and only three were low risk. See Supplementary Figure S1 (available online only) for the complete risk of bias assessment.

## Discussion

This systematic review provides an overview of the acute facility management of blast injuries in LMICs with the goal of serving as a reference for organizational planning and research. In future studies, researchers will be able to compare mortality from blast events with pooled mortality figures, offering a metric by which to interpret the impact of blast events. In the meta-analysis, there were no significant differences in mortality among articles stratified by study country income level and hospital setting. However, both LICs and non-tertiary care center medical facilities are under-represented in the literature, and small sample sizes in these groups may have accounted for the lack of differences.

This meta-analysis showed an overall in-patient mortality of 9.5% and total hospital mortality of 7.4%. Total hospital mortality represents the proportion of hospital deaths, including ED deaths, and is therefore a more accurate representation of the overall mortality of patients affected by blast injuries. In-patient mortality is the proportion of deaths only among admitted patients. Few reviews have calculated pooled in-hospital mortality figures for blast events. In their review of mass-casualty events due to explosive weapons, Arnold, et al reported a pooled ED mortality rate of zero percent and in-patient mortality rate of zero percent to two percent, depending on the type of bombing, a proportion notably lower than the current meta-analysis.^16^ One potential explanation is their inclusion of only large-scale terrorist bombings with 30 or more casualties, events in which a greater proportion of people may have died at the scene as opposed to in the hospital. Furthermore, the studies included were almost exclusively from HICs, where there may be more resources available to manage critically injured patients.

Stratification in the meta-analysis showed that both total hospital mortality and in-patient mortality differed by blast mechanism, with petroleum blasts having significantly higher mortality. The small sample size of three petroleum-related studies limits generalizability. Nonetheless, organizations responding to mass-casualty events due to petroleum explosions should prioritize the availability of specialized burn care and be prepared for potentially higher mortality.

In-patient mortality and total hospital mortality did not differ when stratified by study country income level. This may be due to the small sample size of studies from LICs, which made up less than 15% of included articles, rather than a true equivalence in mortality across country income levels. Under-representation of LICs is likely related to publication bias, wherein publications tend to come from higher income countries with more robust research staff, training, and infrastructure. The broad under-representation of LMICs in research publication is well-documented.^17,18^ The lack of published data from LICs in particular limits the ability to understand conditions like blast injuries that are often seen in these settings. Organizations operating in LICs should implement strategies to strengthen research capacity in order to generate high-quality research in this area and others.

Mortality also did not differ when stratified by hospital setting. This differs from findings by Tovar, et al who determined that terror-related pediatric blast injuries had decreased odds of mortality when treated at a North Atlantic Treaty Organization (NATO; Brussels, Belgium)-affiliated combat hospital.^19^ In this review, well-resourced hospital settings were over-represented with over three-quarters (78.7%) of all studies conducted in either a combat hospital (typically operated by Western military forces) or tertiary care center. The small sample size of studies from less specialized settings, such as community or field hospitals, may have impaired the ability to detect differences in mortality by facility type. Over-representation of high-resource medical facilities also likely impacted reported injury types and interventions because larger medical facilities are more likely to receive complex patients with advanced surgical needs.

Among patients presenting to the ED, the overall rate of in-patient admission was 59.7%. Twenty percent of all admissions were to the ICU. The overall availability of ICU-level care in articles describing a mass-casualty event was 86.5%. This percentage is disproportionate to the availability of critical care in LMICs in general, which is known to be severely lacking, and likely reflects under-representation of publications from LICs and smaller medical facilities in this review. Prior estimates indicate that there are 0.1 to 2.5 ICU beds per 100,000 people in LMICs, compared to 5.0 to 30.0 ICU beds per 100,000 people in HICs.^20^

The assessment of acute interventions and mass-casualty preparedness in this review was limited by what authors chose to mention or quantify within their articles. As a result, substantial conclusions cannot be drawn about triage and surge staffing in blast-related mass-casualty events. The main acute interventions described included IV fluids, antibiotics, and blood transfusion; oxygen therapy and intubation and mechanical ventilation; and emergency surgery. Orthopedic injuries were the most frequently encountered injury type in 74.4% of studies, and limb surgery was the most commonly mentioned acute surgical intervention.^21^ Gastrointestinal, thoracic, and neurologic injuries were also frequently noted. This underlines the importance of multi-specialty care, especially orthopedic surgery, general surgery, and neurosurgery, which is often lacking in resource-limited settings. In contexts where specialized care is not available, streamlined processes to stabilize and expedite transfer of critically injured patients to a higher level of care are essential.

Prehospital care plays a critical role in the triage and initial management of blast injuries. While this topic was not addressed directly by this systematic review, several studies commented on the importance of Emergency Medical Services (EMS) and the effect of prehospital care on facility-based care. Nerlander, et al suggest that long transport times and low quality of prehospital care may have contributed to increased prehospital mortality in their study of victims of blast trauma in Iraq.^22^ Al Hajj, et al reported that 75.5% of patients presenting after the ammonium nitrate blast in Beirut were transported by private vehicle, resulting in a disorganized surge of patients at hospitals.^3^ Efforts to improve emergency preparedness for blast events should emphasize strengthening of EMS systems in conjunction with improvements in facility-level medical care.

## Limitations

There is, in general, a dearth of high-quality studies on the management of blast injuries with most studies (77.3%) being cross-sectional in design. Although randomized-controlled trials are the gold standard in most fields of research, they are likely impractical and even unethical in an emergency or mass-casualty situation. Nonetheless, high-quality quasi-experimental studies examining the effectiveness of different triage methods and emergency interventions would provide a useful evidence base to guide future facility management of blast injuries.

Many of the included studies are of mass-casualty incidents, in which the sudden influx of patients can lead to incomplete or inaccurate charting. As a result, data may be biased. In studies using retrospective data collection, recall bias also limits accuracy and completeness. Furthermore, certain mass-casualty events were more likely to be excluded because injuries occurred by multiple different mechanisms (for example, firearms and explosives) and disaggregated data related specifically to blast injuries were not available. Blast events impacting solely military combatants were excluded, however many studies described civilian injuries related to military action. The authors consciously avoided making summary judgments about whether or not individual studies were conflict-related due to the complexities of these settings.

This systematic review was limited by the heterogeneity in data reporting among published studies, which precluded the authors from conducting a formal meta-analysis of injury epidemiology or interventions. Standardization of data reporting in future research would make it more feasible to compare findings related to interventions and outcomes across publications, permitting more granular insights into the use of specific treatments. Future studies should also consider how outcomes data can be used to evaluate preparedness and quality of care. This review did not include mental health outcomes stemming from blast injuries, which is another topic that warrants further research, particularly in LMICs. Confidence intervals for the secondary outcomes were not adjusted for multiplicity and should be used for hypothesis generation only.

## Conclusion

Blast injuries are medically complex and often occur in the setting of a mass-casualty event, creating further challenges for responders. Skilled triage and stabilization and the availability of specialized care are critical to their successful management. In the meta-analysis, the pooled in-patient mortality was 9.5% and total hospital mortality was 7.4%. There were no significant differences in mortality when stratified by country income level or hospital setting. Low-income countries and non-tertiary care medical facilities are significantly under-represented in the literature.

## Supporting information

Roy et al. supplementary material 1 Roy et al. supplementary material

Roy et al. supplementary material 2 Roy et al. supplementary material

## Supplementary Materials

To view supplementary material for this article, please visit https://doi.org/10.1017/S1049023X25101222
