# Supplementary material for: Acute Facility Management of Blast Injuries In Low- and Middle-Income Countries: A Systematic Review and Meta-Analysis
Source: Prehosp Disaster Med. 2025 Jun 30;40(3):169–81. doi: 10.1017/S1049023X25101222 (PMC12237702; doi:10.1017/S1049023X25101222)
Supplement: Roy et al. supplementary material 2 — Roy et al. supplementary material [file S1049023X25101222sup002.docx]

Table 1. Data extraction template

| Data type | Data abstracted |
| --- | --- |
| Context | Country |
|  | WHO region |
|  | World bank income level |
|  | Hospital setting |
|  | Blast mechanism |
| Interventions | Triage system |
|  | Medications/fluids |
|  | Emergent surgical interventions |
|  | Burn care |
|  | Respiratory support |
| Outcomes | Injury types (by body area) |
|  | Number of ED visits  Number of patients admitted  Number of patients admitted to ICU  Number of patients who had emergency surgery (<24 hours)  Number of patients intubated  Number of deaths in ED  Number of early inpatient deaths (<24 hours)  Number of late inpatient deaths (>24 hours) |
|  | Adverse outcomes |
| Mass casualty response | Triage system in place |
|  | Availability of surge staffing |
|  | Availability of ICU care |
